# British Hospitals Association: The Discussion on Mr. Buchanan's Paper

**Published:** 1915-08-07

**Authors:** 


### The Discussion on Mr. Buchanan's Paper.

The following practical speeches were made:—

Mr. G. Acton Davis, J.P. (treasurer, St. Bartholomew's Hospital), in response to the chairman's invitation, said he had come to learn, and not to offer any views on the point. The difficulty existing in all hospitals was present in St. Bartholomew's also; but Mr. Buchanan had set out the outline of a scheme which had been accepted by St. Bartholomew's, and which would doubtless minimise the difficulties. It was a marvellous change in the administration of hospitals to find that, whereas only a short time ago the students used to, as it were, go down on their knees to obtain resident appointments, it was now the hospitals which had to beg the favour of the acceptance of the important and responsible posts of the hospital. He did not feel able to offer any suggestions to the meeting, as he was not acquainted with the difficulties appertaining to the smaller and non-teaching hospitals. The London Hospital had come to a somewhat similar arrangement to that at St. Bartholomew's, but with some modifications, which seemed to indicate that the London was not as well off for medical officers as his own hospital. He thought the War Office scheme would ensure the satisfactory working of the hospital. It came as news to him that the War Office salaries were so generous. An anæsthetist he knew of was getting fifteen shillings a day for his services, and his rank was that of a major. He believed the difficulties would be got over.

The Secretary (Mr. Thies) read a letter from Mr. Morris, of the London Hospital, detailing the arrangements made between that hospital and the War Office, from which it appeared that the War Office had agreed (1) for all men newly qualified to apply direct to the War Office for a commission, if suitable; and that they should be referred back to the London Hospital, if they had qualified from it, to do three months' practical work in house appointments before being called up. (2) All present holders of house appointments shall be given a commission at once, but shall be liable to be called upon at forty-eight hours' notice when required for training; but not more than five shall be called upon at any one time. (3) That when the War Office so call up the hospital residents they will replace them at the hospital by lending members of the R.A.M.C., qualified men, to fill the vacancies. [Mr. Acton Davis: That is not in our arrangement at all.] These rules were being acted upon. It appeared that eight men were recently taken away, and their places filled by other men who were sent for the purpose. The men who qualified at the hospital in July, numbering about ten, were to do house appointments for at least three months before being required by the War Office. All the residents had been given a commission; and in the last five weeks they had had seven Canadian doctors, of excellent quality, and they were all called back on the 28th ult. Mr. Morris was not now asking for them to be replaced by other men from the War Office, because their own newly-qualified men were filling up the vacancies. At the Horse Guards there was kept a reference-book of doctors in London who were willing to give part-time service to various institutions. The Admiralty had made the same arrangements with the London Hospital.

### The B.M.A. and the Emergency.

Dr. Verrall (representing the British Medical Association) considered Mr. Buchanan's paper a very illuminating one. He came to the meeting because he was chairman of a committee which had been concerned with the war emergency question from the medical point of view in connection with the British Medical Association. His committee was concerned with the shortage of medical officers, and primarily to get doctors for the Army. Their first step was to hold a conference and ask Sir Alfred Keogh to confer with them. He did so, and brought Colonel Blenkinsop. He, the speaker, said to them it was their business to get officers for the Army, without caring much what happened to anybody else. That was admitted. But, he told them, medical men were obliged to consider also hospitals, boards of guardians, and institutions for treating tuberculosis and the like—the case of the ordinary civilian taken ill in private life; hence they could not be expected to take the same view as the War Office. The officers were asked to state what they wanted, then an effort would be made to help them. The opener had said that the advocacy of the position in the *British Medical Journal* had been largely instrumental in getting the men required, but all sorts of sources of supply had been tapped; and now they had come to tap the very freshest supply, the graduates who had just qualified, in that way laying a claim to the very men whom the hospitals wanted. He, the speaker, felt very strongly that if such an attack were made by the War Office it should be made as little harmful as possible. He had, therefore, been very glad to hear the plan of temporary commissions stated. That covered the ground as much as could be expected, because it gave to men a training which would make them, apart from military training, obviously very much fitter people for the Army when they joined it, and it prevented their being taken away before they were wanted. Still, the fact must be faced that if the demand should proceed at the present rate the amount of time which would elapse before that 48-hour notice would be given could not be very great. One heard of men being called up in quantity. His committee had a meeting that day, and they were in touch with the War Office, who promised that the carrying out of their requirements should be made as little harmful to the community as was possible. Still, the need of the Army would be paramount. With regard to the General Medical Council, at a recent meeting the President of that body said he was in process of obtaining fresh relations with Canada and other Colonies so as to give the utmost assistance they could, so that men professionally qualified should not be debarred by matters of what might be designated "red tape"; he hoped to make the conditions still freer. There could be no doubt that the appointment of lady doctors had been a great success. He had not known a case in which a qualified lady had had to be taken in place of a man where the attempt had been a failure. He had attended the meeting so that he might be in a position to make known to his committee what the difficulties were. Nothing in Mr. Buchanan's paper would be forgotten, and anything the members of the British Medical Association could do to minimise the difficulties would be done.

Mr. H. R. Maynard (Secretary, King Edward's Hospital Fund) wished to confirm the interest taken by the King's Hospital Fund in this question. A communication with the War Office ended in that Department suggesting that older men should be utilised by the hospitals. He did not know whether the Fund could do more.

Dr. Cox (Secretary, British Medical Association) said he did not think the help of the general practitioner in the country had been utilised as fully as it might be, either for the War Office or for the civilian population. He was sure there were still general practitioners who, at a pinch, would be willing to do duties at the smaller provincial hospitals. Evidently that was not necessary in the case of hospitals with medical schools attached. His Association had been engaged for some weeks in compiling a War Register, and it enabled them to speak confidently of the majority of the profession—some 80 per cent. in London, and 60 per cent. to 70 per cent. over the whole country—as to what the members were doing and were prepared to do. And it was almost pathetic to see the appeals of men over military age who were yet anxious to be used in some way, and the War Office might very well, he thought, enlist the services of some of these men, though some of the older ones might not be very suitable for the comparatively subordinate work of residentials. Still, many were shedding their ideas of professional and personal dignity, and would be willing to help the hospitals if they knew the extremity to which they were being reduced. He thought that the only other hopeful source of supply was an extension of the system of "seconding" medical officers. One was frequently told that there were many medical men in the Army who were kicking their heels and doing no service. Very divergent views were held about that. The average man who was keen about his business said no man had any business to be kicking his heels; that he could be working twelve hours a day comfortably, and that there was no real wastage. On the other hand, they were told by the War Office that people who were apparently having that leisure were really being kept whence that Department could take them at a moment's notice. He, the speaker, thought it would be possible for the War Office to allow some of the men who at present had not their time fully occupied, and who were not likely to be required to go out of the country for some time, to be turned on to hospital work for a limited time. That seemed quite a reasonable claim to urge on the War Office, and he thought the method operative in London might well be extended to provincial hospitals. He wished to echo what Dr. Verrall had said, that if the British Medical Association, by means of its War Register, could co-operate with the Hospitals Association with the view of obtaining offers for posts in given areas, they would be only too glad to do so.

### The Resolution.

The remaining speakers were Sir William J. Collins, whose points were that women graduates should be increasingly utilised; the removal of the colour bar to enable the graduates of Bombay to be made use of, many of whom were highly valuable and accomplished fellow citizens; and the breakdown of the hard-and-fast line which had sometimes been drawn between the consultant and the medical practitioner; Sir Garrod Thomas, M.D. (Newport); and Dr. James Galloway, who spoke in favour of the employment of Australians and New Zealanders in the London Hospitals.

At the close of the meeting the following resolution was unanimously carried :—

"That the matter be referred to the Executive with a view to securing that all possible sources of supply of resident medical officers are made available, and also to endeavour to bring about co-operation between the War Office, the Admiralty, and the voluntary hospitals in effecting such distribution of available medical officers as may be mutually advantageous."

